# Clinical protocols for ^31^P MRS of the brain and their use in evaluating optic pathway gliomas in children

**DOI:** 10.1016/j.ejrad.2013.11.009

**Published:** 2014-02

**Authors:** Jan Novak, Martin Wilson, Lesley MacPherson, Theodoros N. Arvanitis, Nigel P. Davies, Andrew C. Peet

**Affiliations:** a School of Cancer Sciences, University of Birmingham, Birmingham, United Kingdom; b Birmingham Children's Hospital, Birmingham, United Kingdom; c School of Electronic, Electrical and Computer Engineering, University of Birmingham, Birmingham, United Kingdom; d University Hospitals Birmingham NHS Foundation Trust, Medical Physics RRPPS, Birmingham, United Kingdom

**Keywords:** MR spectroscopy, Chemical shift imaging, MRI, Functional

## Abstract

**Introduction:**

In vivo ^31^P Magnetic Resonance Spectroscopy (MRS) measures phosphorus-containing metabolites that play an essential role in many disease processes. An advantage over ^1^H MRS is that total choline can be separated into phosphocholine and glycerophosphocholine which have opposite associations with tumour grade. We demonstrate ^31^P MRS can provide robust metabolic information on an acceptable timescale to yield information of clinical importance.

**Methods:**

All MRI examinations were carried out on a 3T whole body scanner with all ^31^P MRS scans conducted using a dual-tuned ^1^H/^31^P head coil. Once optimised on phantoms, the protocol was tested in six healthy volunteers (four male and two female, mean age: 25 ± 2.7). ^31^P MRS was then implemented on three children with optic pathway gliomas.

**Results:**

^31^P MRS on volunteers showed that a number of metabolite ratios varied significantly (*p* < 0.05 ANOVA) across different structures of the brain, whereas PC/GPC did not. Standard imaging showed the optic pathway gliomas were enhancing on T1-weighted imaging after contrast injection and have high tCho on ^1^H MRS, both of which are associated with high grade lesions. ^31^P MRS showed the phosphocholine/glycerophosphocholine ratio to be low (<0.6) which suggests low grade tumours in keeping with their clinical behaviour and the histology of most biopsied optic pathway gliomas.

**Conclusion:**

^31^P MRS can be implemented in the brain as part of a clinical protocol to provide robust measurement of important metabolites, in particular providing a greater understanding of cases where tCho is raised on ^1^H MRS.

## 1 Introduction

In vivo Magnetic Resonance Spectroscopy (MRS) is able to profile the chemical composition of defined regions of interest in the brain. The ^1^H nucleus is the most commonly probed due to its high gyromagnetic ratio and widespread development of both hardware and techniques. While the use of ^1^H MRS is now becoming more prevalent for brain tumour diagnosis and assessment of treatment efficacy, it can be difficult to implement across some important regions of the brain and cannot reliably quantify a number of metabolites of clinical and biological importance [1]. ^31^P MRS can measure a number of these key metabolites, does not suffer from problems of water and fat contamination and requires a less homogeneous magnetic field. Prior to the development of water suppression, ^31^P was the nucleus of choice for investigating in vivo metabolic activity but has since suffered from a lack of technical development and integration into clinical MRI systems. ^31^P MRS may seem unattractive due to the low SNR which leads to poor spatial resolution (large voxel sizes) and importantly long acquisition times [2]. Other barriers to its widespread use are the requirement of a dedicated ^31^P coil and the lack of advanced processing software available for automated quantification of the metabolites. However, technical advances are making ^31^P MRS a viable option clinically and it is now appropriate to develop and evaluate protocols suitable for clinical practice and use them to investigate brain metabolism.

Higher field scanners provide increased SNR [3], reducing scan times, dedicated head coils have recently become available, providing complete brain coverage [4] and the advent of ^1^H-decoupled ^31^P MRS has increased the spectral resolution allowing phosphomonoesters (PME) and phosphodiesters (PDE) to be deconvoluted into their constituent resonances. Phosphocholine (PC), glycerophosphocholine (GPC), glycerophosphoethanolamine (GPE) and phosphoethanolamine (PE) can be separately identified overcoming a limitation of clinical ^1^H MRS which can only detect a tCho peak, which is a linear combination of these metabolites. In tumours, it is well established that the biomarker roles of PC and GPC are different and this could account for the conflicting results of studies on the relationship between tCho and tumour aggressiveness [5]. A recent study has shown the importance of PC and GPC levels in differentiating Grade II and IV Astrocytomas using ex vivo MRS in adults [6] where elevated levels of PC were found in the Grade IV lesions and high GPC was found in the Grade II lesions. There is also evidence that the ratio PC/GPC could be a useful biomarker of tumour response [7]. One clinical scenario where this technique could be particularly important is paediatric optic pathway gliomas. These are often large, unresectable tumours which are enhancing with a high tCho peak. Ordinarily, this would be indicative of a high grade aggressive tumour but if biopsied they are commonly diagnosed as grade I pilocytic astrocytomas. These tumours present major challenges for clinical management since their behaviour is varied and cannot be predicted by conventional techniques. A non-invasive tool for accurate diagnosis, prognosis and monitoring would be a major advance. It is hypothesised that deconvolution of the high tCho peak could provide further understanding of the biology of these tumours and yield important new biomarkers. ^31^P MRS has other potential uses including evaluation of high-energy phosphates, adenosine-triphosphate (ATP) and adenosine diphosphate (ADP) and measures the pH of a system using relative shift of measured peaks [8]. The development of a ^31^P MRS method which provides information on the key phosphorous-containing metabolites on a clinically applicable timescale from any region in the head would be a major step forward in the implementation of the technique as a tool for radiologists.

In this study we develop a ^31^P MRS protocol in phantoms and volunteers and then adapt it to the investigation of regional metabolite variability across the brain in healthy adult volunteers and to paediatric patients with unbiopsied optic pathway glioma. Quantification of the spectra is facilitated by a new adaptation of the fully automated TARQUIN algorithm [9] to ^31^P MRS.

## 2 Materials and methods

### 2.1 Phantom design

A phantom was designed to assess the signal-to-noise ratio, decoupling and localisation efficiency of ^31^P MRS. This consisted of a cylindrical plastic container in which the bottom half was filled with a 1% (w/w) agarose gel (Invitrogen Life Technologies) with 5 mM sodium phosphate (Sigma Aldrich). This layer was then sealed with a non-permeable polymer film. Once solidified, another layer of 1% agarose gel was poured on top, this time with 5 mM phosphoethanolamine (Sigma Aldrich).

### 2.2 MR acquisition

All data were acquired on a Philips Achieva 3 T TX system (Best, Netherlands). Volunteer and phantom scans were conducted solely using a dual-tuned ^31^P/^1^H head coil (Rapid Biomedical, Germany). Parameters for the final acquisition protocol used in patients were optimised using a combination of phantoms and volunteers.

Patient data were acquired using a combination of both the ^31^P/^1^H head coil and a 32 channel ^1^H Philips head coil. Standard imaging was performed using the 32 channel ^1^H head coil or the neurovascular coil. Basic localiser imaging and ^31^P spectroscopy was performed using the ^31^P/^1^H head coil.

### 2.3 Phantom experiments

A three plane T1-weighted localiser imaging sequence was used for spectroscopic volume positioning. Prior to the acquisition of ^31^P spectra, a single slice 10 mm thick T1-weighted image was acquired in the same orientation as the ^31^P MRS acquisition to enable accurate positioning and post-experimental analysis.

### 2.4 Protocol optimisation on volunteers

Further optimisation of parameters was conducted using volunteers. Voxels were 30 mm × 30 mm with a slice thickness of 30 mm resulting in a volume of 27 cm^3^. Improved SNR and equivalent shim was observed for 35 mm × 35 mm voxels which were employed in patients when tumours were sufficiently large. The echo time was the shortest possible at 0.31 ms. To ensure optimal spatial localisation using the ISIS technique, eight averages were acquired. The bandwidth was 3000 Hz with 1024 spectral points collected.

**Fig. 1:**
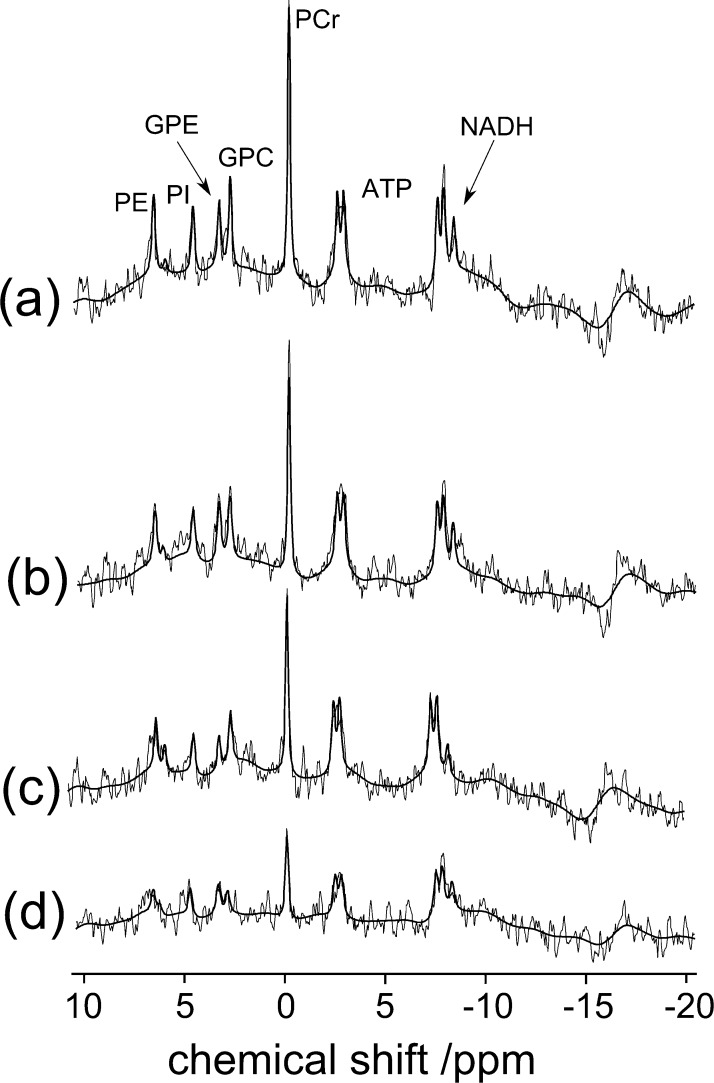
A series of ^31^P MR spectra acquired from a mixture of white and grey matter with different repetition times (TRs). The feint lines represent the raw data and the dark line represents the fit. (a) TR = 4000 ms, (b) TR = 3000 ms, (c) TR = 2000 ms and (d) TR = 1000 ms.

The repetition time was optimised via a series of scans which were conducted on the white/grey matter of volunteers at four different TRs. Representative data from these scans are shown in Fig. 1. It was found that increasing the TR gave a greater improvement in the SNR than would have been achieved by increasing the number of averages for the same total acquisition time. It was decided that a TR of 4000 provided an appropriate level of SNR and still would be able to measure signals from the choline-containing metabolites in less cellular tumours which may have lower concentrations. TRs < 4000 resulted in insufficient SNR and TRs < 3000 ms introduced T1 saturation effects.

### 2.5 Volunteer studies using the optimised protocol

Six healthy volunteers (four male and two female) were studied with a mean age of 25 ± 2.7 in full compliance with our research ethics.

**Fig. 2:**
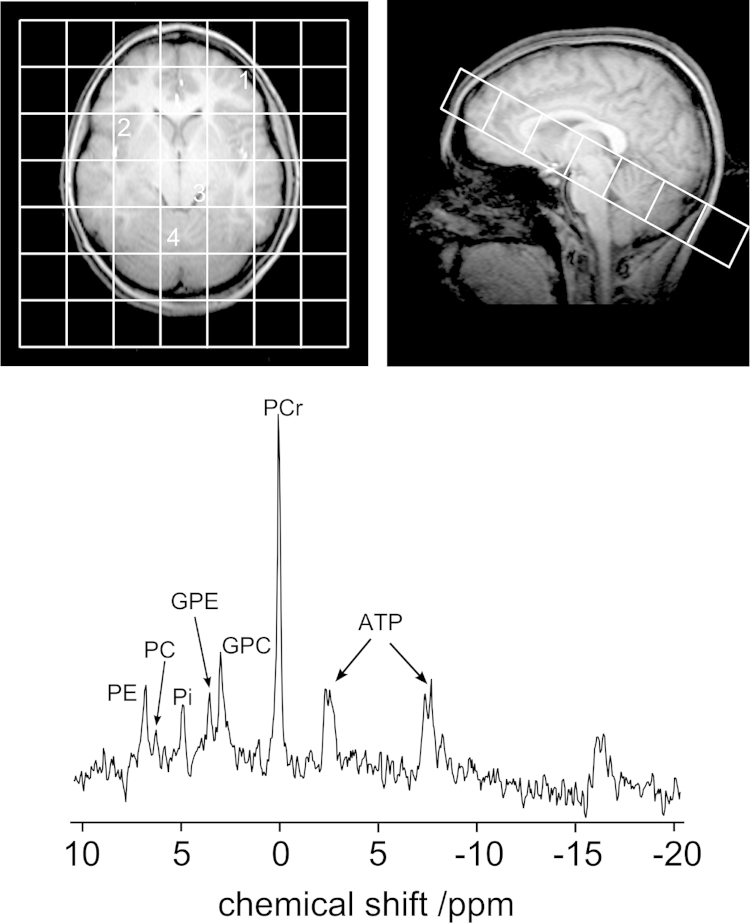
Representative ^31^P Magnetic Resonance Spectroscopic Imaging (MRSI) spectrum obtained from a healthy volunteer. The orientation of the pseudo axial image shown on the left is the same orientation as the MRSI grid shown on the right hand side. The orientation of the MRSI grid allowed simultaneous metabolic profiling of various regions of the brain: the cerebellum (voxel 4), the brain stem (voxel 3), the basal ganglia (voxel 2) and the white/grey matter of the frontal lobe (voxel 1). The representative ^31^P spectrum was obtained from the brain stem.

Fig. 2 shows the positioning of the MRSI grid used to acquire the ^31^P spectra in volunteers. The regions actively targeted for this study were the basal ganglia, which is mainly grey matter and is of particular interest for inherited metabolic disorders [10], the frontal lobe which contains a mixture of white and grey matter, the brain stem and the cerebellum. A single MRSI slice was sufficient to capture all of the aforementioned regions, although all voxels contained multiple tissue types due to the large size. The ^31^P MRS acquisition time for the volunteer study was 20 min using a MRSI grid size of 7 × 7. The ^31^P MRS parameters for both the volunteer study and the proposed protocol for patients are summarised in Table 1.

### 2.6 Patient acquisitions

Three patients (two male and one female, age = 2.7 ± 1.5 years) with unbiopsied, optic pathway gliomas were investigated. Informed parental consent was obtained prior to the ^31^P MRS examination which was conducted after essential clinical imaging. Post clinical imaging, the ^1^H head coil was removed from the scanner and replaced with the dual-tuned ^1^H/^31^P head coil. ^31^P scanning time varied between patients, with the longest time 12 min and the shortest 6 min.

Single voxel ^1^H spectra (SVS) were acquired using the Point REsolved SpectroScopy (PRESS) technique employing a short echo time (37–40 ms). The voxels were 2 cm × 2 cm × 2 cm (8 cm^3^ volume) for all of the cases. A repetition time of 2000 ms was used with 96–128 averages acquired. The bandwidth was 2000 Hz with 1024 data points acquired with a total scanning time of 6 min.

### 2.7 Processing and analysis

All data were exported from the scanner in DICOM format and subsequently processed by version 4.2.6 of the TARQUIN algorithm [9] for the volunteers, version 4.2.9 was used for phantoms and version 4.2.10 was used for patients. These versions of TARQUIN have been adapted specifically to allow analysis of ^31^P MRSI data with the latter versions allowing manual phasing and peak referencing which is ideal for phantom studies. The ^31^P spectra for volunteers were automatically processed by TARQUIN including Fourier transformation, zeroth and first order phasing and fitted using a simulated ^31^P brain basis set. Prior to fitting, all spectra were referenced to PCr which was set to 0 PPM on the chemical shift scale.

New basis sets were optimised for both the volunteers and the brain tumour patients. The following metabolites were simulated using a single Lorentzian peak PE, PC, PI_(tissue)_ (inorganic phosphate in the tissue), GPE, GPC, PCr, and NADH. ATP was simulated using two of the 3 resonances: γ and α peaks which were both doublets with a j-coupling of 16 Hz. β-ATP was excluded from the basis set due to phasing issues. Despite being a superposition of ATP and ADP peaks, the resonances will be referred to only as ATP. Two additional peaks were included in the basis set for the brain tumour patients which were: PI_(blood)_, which is inorganic phosphate found in the blood and a peak at 2.2 PPM which has been previously attributed to phosphoenol pyruvate (PEP) [11].

All spectra were assessed by an experienced spectroscopist and quality assessment was applied to the spectroscopy. All of the ^31^P data passed QA apart from one spectrum in the frontal lobe.

The absolute quantification of metabolites measured using ^31^P spectroscopy is challenging due to the lack of water reference which can be used as a denominator. No corrections have been made for the different ^31^P T1 values for metabolites within the brain due to the long times associated with accurate T1 measurements. Multiple TR scans indicated that major differences did not exist between the T1 values of the different metabolites. No corrections were made for different T2 values for various metabolites measured by ^31^P experiments or for the NOE enhancement of separate resonances.

### 2.8 Statistics

All statistical calculations were conducted using the program R, version 2.15.0 (the R foundation for Statistical Computing). For the ratios, the variance was analysed using an ANOVA test across the four different regions of the brain. If the ANOVA test revealed a statistical difference (*p* < 0.05) a Tukey's significant difference test was performed to allow for multiple comparisons.

## 3 Results

### 3.1 Phantom optimisation

**Fig. 3:**
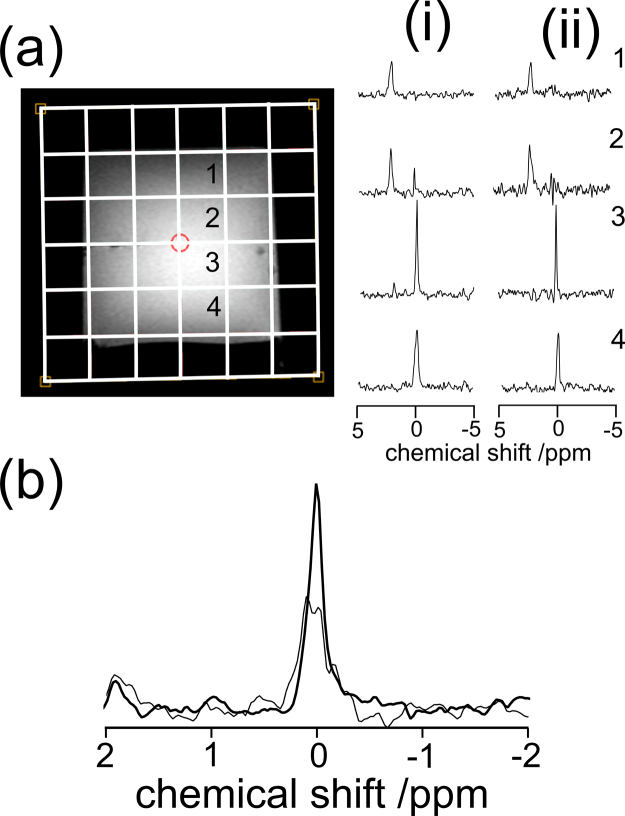
(a) Shows a localiser image in an axial orientation of a gel phantom filled with 1% agar. Voxels 1 and 2 are placed over the gel containing inorganic phosphate (PI) and voxels 3 and 4 were placed over gel containing phosphoethanolamine (PE). Spectra 1–4 correspond to the voxels in the image with the elliptical *k*-space mask on in (i) and off in (ii). The peak at 0 ppm corresponds to PE and the peak at 2 ppm corresponds to PI. (b) Shows the effect of decoupling on the PE resonance in the gel phantom. The thicker line shows the PE resonance with decoupling and the thinner line is without decoupling.

The images for the phantom and spectra showing both localisation and decoupling for the phantoms are shown in Fig. 3. ^31^P spectra were acquired using a 2D Magnetic Resonance Spectroscopic Imaging (MRSI) technique employing Image Selected In vivo Spectroscopy (ISIS) localisation. The data acquired for the localisation efficiency is shown in Fig. 3(a). The effect of an elliptical mask, which samples 80% of *k*-space, therefore reducing the acquisition time, was investigated using the gel phantom described above and is also demonstrated in Fig. 3(a). An inter-voxel signal contamination of 7–11% in the absence of the filter and 18–25% with the filter was found.

Broadband decoupling was carried out using the ^1^H channel of the dual tuned coil using a WALTZ4 technique with a frequency offset of −100 Hz. The decoupling duration was limited to 237.2 ms instead of the optimal 347.7 ms because of hardware constraints. This did not visibly affect decoupling efficiency due to the short T2 values of the ^31^P nuclei which result in a rapid signal decay. Phantom data in Fig. 3(b) shows the decoupling was able to resolve the PE resonance from a triplet to a singlet, effectively reducing the line width of the PE peak from 19 to 9 Hz. The decoupling efficiency was also evaluated in volunteers showing clearly resolved peaks for PE, PC, GPE and GPC.

Narrowband NOE enhancement was used to further increase the SNR using an offset of −100 Hz and a mix time of 3500 ms and a B1 max of 0.3 μT. Phantom measurements showed that the signal intensity was increased by 6% for PE and 5% for inorganic phosphate.

### 3.2 Volunteer metabolite ratios

The collated ratio data from all regions of the brain for ^31^P metabolites are shown in Table 2 which indicated metabolite ratios vary throughout the brain. A significantly lower PCr/ATP ratio was found in the basal ganglia when compared to both the brain stem (*p* < 0.05) and the cerebellum (*p* < 0.005). PCr/ATP was significantly higher in both the brain stem and the cerebellum compared with the frontal lobe (both *p* < 0.05). GPE/ATP ratio was found to be higher in the brain stem than in the basal ganglia (*p* < 0.05). Significantly higher PE/GPE was found in both the basal ganglia (*p* < 0.05) and the frontal lobe (*p* < 0.05) compared to the brain stem. Lower values for PE/PCr were found in the brain stem compared to the basal ganglia (*p* < 0.05) and the frontal lobe (*p* < 0.01). Lower PE/PCr was also found when spectra from the cerebellum were compared to both the basal ganglia (*p* < 0.05) and the frontal lobe (*p* < 0.005). There was no significant difference detected in the PC/GPC ratio across the brain regions evaluated. The study would have had 80% power to have detected a difference of 0.16 in the PC/GPC ratio with a confidence level of 5% (2 tailed *T*-test).

### 3.3 Patient data

**Fig. 4:**
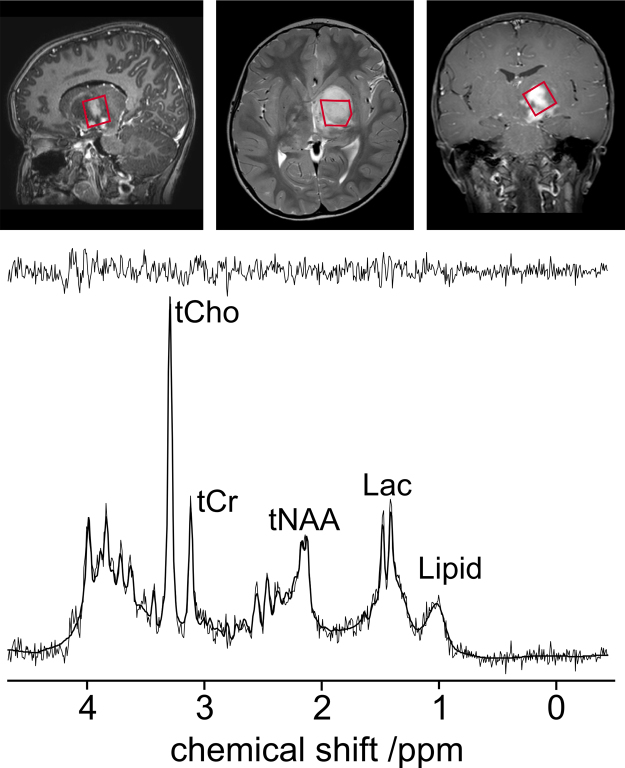
Showing the positioning of a 2 cm × 2 cm × 2 cm single-voxel ^1^H Magnetic Resonance Spectroscopy (MRS) acquisition of an unbiopsied optic pathway glioma and below it the ^1^H MR spectrum acquired. The lighter line in the ^1^H MR spectrum is the data and the solid black line is the fit from the TARQUIN software.

A representative ^1^H MRS PRESS single voxel spectrum acquired from a paediatric optic pathway glioma is shown in Fig. 4. The important feature of this spectrum is the exceptionally high tCho relative to the other metabolites in the spectrum. The tNAA is relatively low, indicating low neuronal density which is consistent with brain tumours. The presence of both lipids and lactate is consistent with brain lesions and is not ordinarily found in healthy brain tissue.

**Fig. 5:**
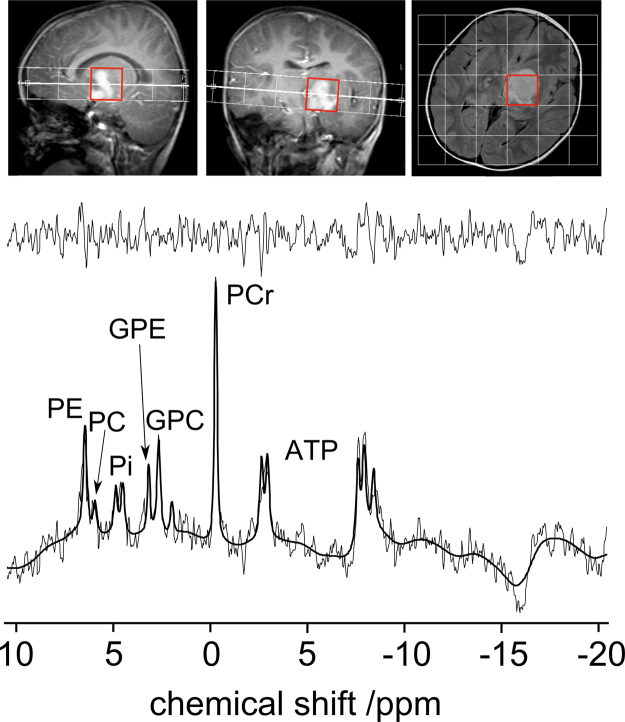
Showing a ^31^P Magnetic Resonance Spectroscopic Imaging (MRSI) acquisition of a paediatric patient with an unbiopsied optic pathway glioma. The displayed spectrum was obtained from the voxel highlighted in red. (For interpretation of the references to colour in this figure legend, the reader is referred to the web version of the article.)

A ^31^P MRSI spectrum of the same paediatric optic pathway glioma is shown in Fig. 5 along with the MRSI grid positioning. The quality of the spectra was good and importantly the spectral separation of the choline-containing peaks was even better than observed in healthy volunteers. The PCr peak was lower than was observed for the volunteers which is consistent with the ^1^H spectroscopy where total creatine was low compared to healthy brain. The PI peak is no longer represented by a single peak but has split into two resonances. The upfield peak (PI_(tissue)_) is consistent with brain tissue (pH calculated as ≈7) and the downfield peak(PI_(blood)_) consistent with blood where the pH is calculated as ≈7.35.

Significant PEP levels were observed in one patient. The PC/GPC ratio is low across all of the patients and all other metabolite ratios are shown in Table 3. The table also includes internal control ratios from the patients where uninvolved brain is included in the MRSI grid. This data was derived from an average of voxels which do not include tumour on the conventional MRI and were chosen for both spectral quality and distance from the lesion. An average from different anatomical regions was used since the volunteer data showed that there was no statistical difference present for PC/GPC across different brain regions (at least in young adults). We were unable to use purely contralateral voxels as the tumours are positioned in the midline of the brain or affect both hemispheres.

## 4 Discussion

A ^31^P MRS imaging protocol has been developed using a dedicated head coil and implemented in volunteers to obtain metabolite values across a range of different tissues in the brain. The method was straightforward to implement and produced good quality data in all volunteers, despite selecting a challenging volume of interest which included the cerebellum, brain stem, basal ganglia and frontal cortex. Data acquisition was combined with a new technique for the automated quantification of metabolite levels in ^31^P MRS to provide a straightforward method for obtaining ratios of phosphorous-containing metabolites. Metabolite ratios were quantified accurately enough to observe significant variations between different regions of the brain. The protocol can be adapted to be used on a clinically viable timescale without any detrimental effect on the quality of the data, which is crucial if it is to be used on patients.

Variation in the concentration of metabolites within different regions of the brain has been studied previously using ^1^H MRS [12] which indicated that metabolites are not homogeneously distributed across all structures within the brain. A study by Buchli et al. compared ^31^P-measured metabolites in cortical brain and the cerebellum of healthy volunteers [13]. They showed there were metabolite concentration differences between both the cerebrum and the cerebellum and also the deep white and cortical grey matter. A paper studied metabolites in diseased and healthy midbrain and the putamen using ^31^P MRS [14] but no comparisons between brain regions were made. To our knowledge, the variation of metabolites across multiple structures in the brain within the same subject has not been systematically studied using ^31^P MRS. Whilst the number of volunteers in the study is relatively small, the variability between the subjects is sufficiently low that the most important findings are likely to have been detected and be reproducible. However, it would be worthwhile confirming the findings in a larger cohort which would also have the power to detect more subtle differences between brain regions.

Our experience is that the technique was easier to implement and more robust (supplementary figure) than ^1^H MRSI, where the collection of data across the investigated regions was very challenging. Methods previously used for quantification of ^31^P MRS include phantom replacement [14] and in situ phantom calibration [15] both of which are impractical in a busy clinical environment. For this study we used solely metabolite ratios, almost all of which measured in the volunteers were within the reported errors of the control data from children published in the paper by Bluml et al. [16]. Their study did not employ a dual-tuned head coil with full head coverage and was conducted using a field strength of 1.5 T. Our use of a dedicated head coil allows the study of multiple regions of the brain and utilising field strength of 3 T affords greater SNR per unit time. It is encouraging that we observe similar ratios to those reported in the study by Bluml et al. despite the differences in the acquisition protocol and the hardware used to acquire the data. The only observed differences between the two studies were in the metabolite PE which can be explained by the relative ages of the controls used by Bluml et al. and our volunteers. Our volunteers were significantly older (25 ± 2.7) than the Bluml study (6.3 ± 5.3) and PE has been shown to significantly decrease as a function of age [17].

Supplementary material related to this article can be found, in the online version, at http://dx.doi.org/10.1016/j.ejrad.2013.11.009.

**Supplementary Figure S1:**
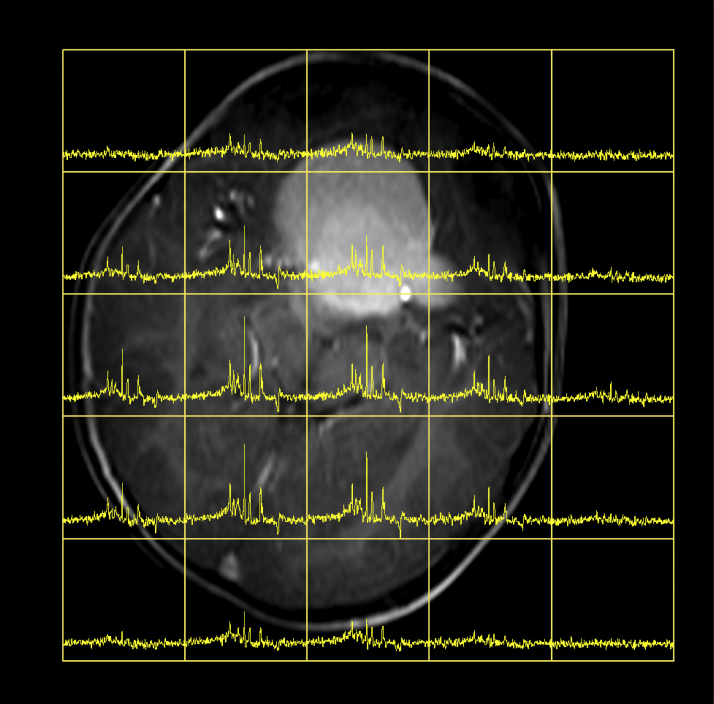
Showing ^31^P MRSI spectra overlaid on an axial T1 weighted image for a patient with an optic pathway glioma. The spectra are high quality throughout the brain even across the brain/skull interfaces demonstrating the robustness of the technique. Lower SNR is seen in the voxels which are only partly filled with tissue as expected.

^1^H MRS measurements for the optic pathway gliomas all show high total choline relative to the other metabolites. This phenomenon is counter intuitive when compared with the general finding that high tCho is indicative of high malignancy [5]. The tCho peak is composed mainly of PC and GPC and it has been shown previously that the PC/GPC ratio is a better indicator of tumour aggressiveness with PC dominating in high grade tumours but GPC being high in those of low grade [16,18], although it is not yet certain which characteristics associated with malignancy the PC/GPC ratio best reflects particularly for paediatric tumours. When assessed with ^31^P MRS, we have shown that the majority of the tCho peak is accounted for by GPC as opposed to PC, confirming the low grade nature of these tumours. We do not believe that the PC/GPC ratio can be attributed to age related changes in these metabolites as very little difference has been observed above one year of age [17]. There could be some contribution to the tCho peak from free choline which would not be detected by ^31^P MRS but levels of this metabolite are usually low in tumours and it has not been linked to tumour grade [18]. There are also contributions to the tCho peak from PE and GPE but these are low due to the comparatively small number of protons contributing to the peak at 3.2 ppm (1 for PE and GPE and 9 for GPC and PC) and the J-coupling which effectively reduces the peak height. The low PC/GPC ratio measured in the optic pathway gliomas is consistent with the low grade nature of these tumours and we believe is the only known non-invasive biomarker which provides this information on this type of tumour. The technique used is particularly pertinent to tumours which are located deep within the brain for which surgery poses a significant risk such as the cases presented here. These findings warrant the study of a larger patient cohort to evaluate PC/GPC and other ^31^P MRS biomarkers in the diagnosis, prognosis and treatment efficacy monitoring of these tumours.

## 5 Conclusion

^31^P MRS is easy to acquire with a dedicated head coil and produces robust and reproducible results across a number of important brain regions in a clinically applicable timescale. Differences in metabolite ratios occur across healthy brain and need further investigation. Metabolite levels in volunteers are consistent with those previously reported, implying that ^31^P MRS is reproducible across different scanners. ^31^P MRS in optic pathway gliomas of childhood exhibiting, high tCho peaks in ^1^H MRS, showed that GPC contributed more to this peak than PC. We believe that the PC/GPC ratio is the first non-invasive biomarker showing these tumours are low grade. ^31^P MRS should be more extensively used in the assessment of brain tumours and its optimum role in clinical practice determined.

## Conflict of interest

There is no conflict of interest for the work submitted in this document.

## Figures and Tables

**Table 1:** Showing the finalised ^31^P protocol for both the volunteer study and the protocol used for the patients.

Parameter	Volunteer study	Patient protocol
Localisation	MRSI (ISIS)	MRSI (ISIS)
*k*-Space mask	Elliptical (80%)	Elliptical (80%)
TR/ms	4000	3000–4000
TE/ms	0.31	0.31
Number of averages	8	8
Voxel size/mm	30 × 30	≤35 × 35
Slice thickness/mm	30	30
Matrix size	7 × 7	≥6 × 5
Bandwidth/Hz	3000	3000
Data points acquired	1024	1024
Acquisition time/min	20	6–12

**Table 2:** Showing collated data from the volunteers. The data are reported as the mean ± the standard deviation.

Metabolites	Brain stem	Cerebellum	Basal ganglia	Frontal lobe	ANOVA test *p*
PE/ATP	0.56 ± 0.05	0.56 ± 0.05	0.59 ± 0.11	0.63 ± 0.10	0.429
GPE/ATP	0.51 ± 0.07	0.43 ± 0.06	0.39 ± 0.08	0.42 ± 0.08	0.047*
PC/ATP	0.23 ± 0.04	0.22 ± 0.05	0.16 ± 0.07	0.14 ± 0.14	0.211
GPC/ATP	0.66 ± 0.09	0.58 ± 0.10	0.56 ± 0.12	0.61 ± 0.10	0.453
PI/ATP	0.46 ± 0.11	0.41 ± 0.05	0.40 ± 0.09	0.45 ± 0.09	0.473
PCr/ATP	1.72 ± 0.16	1.80 ± 0.20	1.34 ± 0.11	1.38 ± 0.30	0.001*
NADH/ATP	0.25 ± 0.05	0.26 ± 0.10	0.23 ± 0.10	0.38 ± 0.09	0.062
PE/GPE	1.11 ± 0.17	1.32 ± 0.22	1.55 ± 0.33	1.53 ± 0.15	0.013*
PC/GPC	0.35 ± 0.05	0.39 ± 0.10	0.28 ± 0.12	0.25 ± 0.25	0.380
PC/PE	0.40 ± 0.06	0.40 ± 0.08	0.27 ± 0.13	0.23 ± 0.23	0.118
PE/PCr	0.33 ± 0.02	0.31 ± 0.03	0.44 ± 0.07	0.47 ± 0.11	0.0009*

* Indicates a significant difference was present (*p* < 0.05).

**Table 3:** Showing metabolite ratios for both the tumours and healthy tissue of three paediatric patients with optic pathway gliomas. The normal data for each patient represent the mean of voxels with normal appearing brain on conventional MRI included in the MRSI grid. The ± value is the standard deviation.

Metabolites	Patient 1		Patient 2		Patient 3	
	Normal	Tumour	Normal	Tumour	Normal	Tumour
PC/GPC	0.60 ± 0.18	0.41	0.48 ± 0.17	0.34	0.49 ± 0.12	0.57
GPE/ATP	0.31 ± 0.05	0.22	0.25 ± 0.04	0.31	0.23 ± 0.00	0.14
PC/ATP	0.25 ± 0.05	0.19	0.21 ± 0.07	0.20	0.17 ± 0.05	0.21
GPC/ATP	0.45 ± 0.15	0.47	0.45 ± 0.04	0.59	0.35 ± 0.04	0.38
PI/ATP	0.23 ± 0.08	0.24	0.30 ± 0.07	0.31	0.25 ± 0.02	0.18
PCr/ATP	1.20 ± 0.04	0.82	1.00 ± 0.07	1.21	1.06 ± 0.07	0.71
NADH/ATP	0.38 ± 0.04	0.42	0.33 ± 0.02	0.35	0.34 ± 0.05	0.26
PE/GPE	2.04 ± 0.54	2.52	2.59 ± 0.37	2.14	2.64 ± 0.17	4.50
PC/PE	0.40 ± 0.04	0.36	0.34 ± 0.11	0.30	0.28 ± 0.07	0.33
PE/PCr	0.52 ± 0.04	0.66	0.63 ± 0.04	0.55	0.56 ± 0.03	0.91
